# Nitric Oxide Alters the Pattern of Auxin Maxima and PIN-FORMED1 During Shoot Development

**DOI:** 10.3389/fpls.2021.630792

**Published:** 2021-04-26

**Authors:** Inmaculada Sánchez-Vicente, Tamara Lechón, María Fernández-Marcos, Luis Sanz, Oscar Lorenzo

**Affiliations:** Departamento de Botánica y Fisiología Vegetal, Instituto Hispano-Luso de Investigaciones Agrarias (CIALE), Facultad de Biología, Universidad de Salamanca, Salamanca, Spain

**Keywords:** auxin response, auxin transport, leaf morphology, nitric oxide homeostasis mutants, PIN-FORMED 1

## Abstract

Hormone patterns tailor cell fate decisions during plant organ formation. Among them, auxins and cytokinins are critical phytohormones during early development. Nitric oxide (NO) modulates root architecture by the control of auxin spatial patterns. However, NO involvement during the coordination of shoot organogenesis remains unclear. Here, we explore the effect of NO during shoot development by using a phenotypic, cellular, and genetic analysis in *Arabidopsis thaliana* and get new insights into the characterization of NO-mediated leaf-related phenotypes. NO homeostasis mutants are impaired in several shoot architectural parameters, including phyllotactic patterns, inflorescence stem elongation, silique production, leaf number, and margin. Auxin distribution is a key feature for tissue differentiation and need to be controlled at different levels (i.e., synthesis, transport, and degradation mechanisms). The phenotypes resulting from the introduction of the *cue1* mutation in the *axr1* auxin resistant and *pin1* backgrounds exacerbate the relationship between NO and auxins. Using the auxin reporter *DR5:GUS*, we observed an increase in auxin maxima under NO-deficient mutant backgrounds and NO scavenging, pointing to NO-ASSOCIATED 1 (NOA1) as the main player related to NO production in this process. Furthermore, polar auxin transport is mainly regulated by PIN-FORMED 1 (PIN1), which controls the flow along leaf margin and venations. Analysis of PIN1 protein levels shows that NO controls its accumulation during leaf development, impacting the auxin mediated mechanism of leaf building. With these findings, we also provide evidence for the NO opposite effects to determine root and shoot architecture, in terms of PIN1 accumulation under NO overproduction.

## Introduction

Leaf initiation from the shoot apical meristem (SAM), and later development are controlled by networks composed by plant hormones and transcriptional factors, which determine organ form and size. Leaf shape and size change between different species and around an individual plant. Although some variation exists depending on environmental circumstances and internal conditions, main signaling mechanisms surrounding these processes are influenced by spatial patterns based on auxin gradients (Avsian-Kretchmer et al., 2002; Heisler et al., 2005; Cheng et al., 2007; Marcos and Berleth, 2014). Specific points of auxin maxima are described previously to leaf initiation, involving changes in the polar localization of efflux carriers [i.e., PIN-FORMED (PIN1) proteins]. PIN transporters modulate this auxin flow in a highly controlled manner in order to organize cell and organ growth (Reinhardt et al., 2000, 2003; Benková et al., 2003; Qi et al., 2014).

During the regulation of physiological and stress processes, the gasotransmitter nitric oxide (NO) usually interacts with plant hormones and other endogenous molecules, affecting their biosynthesis, catabolism, transport, perception, and signal transduction (reviewed in Freschi, 2013; Sanz et al., 2015). Even though NO synthesis in plants is still partially unresolved, the best characterized mechanisms involve the reduction of nitrite by NITRATE REDUCTASES 1 (NIA1) and 2 (NIA2) and the oxidation in an arginine-dependent pathway by NO-ASSOCIATED 1 (NOA1), similar to the animal NO synthase (NOS) activity (reviewed in Astier et al., 2018). There is plenty of evidence that NO acts downstream of auxin (just in *A. thaliana*, Lombardo et al., 2006; Flores et al., 2008; Kolbert et al., 2008; Kolbert and Erdei, 2008; Chen et al., 2010; Wang et al., 2010; Schlicht et al., 2013; Cao et al., 2017; Fattorini et al., 2017; Moro et al., 2017; Sun et al., 2017; Liu et al., 2018), but several reports have shown that mutants which overproduce NO or the addition of exogenous NO also increase auxin levels (Xu et al., 2010; Chen et al., 2013; Elhiti et al., 2013; Manoli et al., 2015; Wen et al., 2016). However, the role of NO in auxin accumulation seems to be indirect. The use of NO donors in *Medicago truncatula* seedlings under cadmium stress improves stress tolerance by reducing indoleacetic acid (IAA) oxidase-driven auxin degradation, thus maintaining auxin equilibrium (Xu et al., 2010). Furthermore, knocking out phytoglobins (PGBs), which increase endogenous NO levels, inhibits auxin metabolism, resulting in a drastic modification of embryogenesis and root development (Elhiti et al., 2013).

Since NO had been described to act downstream of auxin, especially during root hair (Lombardo et al., 2006) and lateral root formation (Wang et al., 2010; Cao et al., 2017), Terrile et al. (2012) demonstrated that it might do so through promotion of auxin signaling *via S*-nitrosation of TIR1, which would enhance the interaction between SCF^TIR1^ and the Aux/IAAs, facilitating their degradation. However, previous reports had shown that, even though pharmacological NO donors increased lateral root formation and inhibited primary root development, they did not increase auxin response, since they could not elicit auxin-responsive promoters *DR5::uidA* and *BA3::uidA* (Méndez-Bravo et al., 2010; Pető et al., 2011). This was later confirmed using NO-overproducer mutants (Fernández-Marcos et al., 2011) and since then, several independent researchers have shown that AXR3/IAA17, one of the Aux/IAA transcriptional repressors, is stabilized by NO (Shi et al., 2015, 2017; Yuan and Huang, 2016). In addition, NO represses *TIR1* transcription (Vitor et al., 2013), so it seems that the interaction between NO and auxin is mostly antagonistic. The observed reduction in auxin response caused by high levels of NO might be explained by a reduction in polar auxin transport (Fernández-Marcos et al., 2011), and NO homeostasis mutants present alterations in auxin transport (Fernández-Marcos et al., 2011; Sanz et al., 2014; Shi et al., 2015), an effect that can be replicated using a pharmacological approach (Adamakis et al., 2014; Sanz et al., 2014; Yuan and Huang, 2016; Sun et al., 2017; Liu et al., 2018). Furthermore, it has been shown that GSNO affects the polar distribution of PIN2 in roots during gravitropism (Ni et al., 2017; París et al., 2018).

To extend our understanding of the effect of NO during shoot organization and leaf determination, we have studied the phenotype of NO homeostasis mutants, highlighting an altered leaf phenotype, including size and number of leaves and structural characteristics like the number of serrations. We also detected defects related to shoot architectural parameters, including phyllotactic patterns, inflorescence stem elongation, and silique production. In order to deepen on the NO and auxin crosstalk, we studied the weakened phenotypes resulting from the introduction of *cue1* mutation in *auxin resistant 1 (axr1)* background (a mutant with impaired auxin signaling since AXR1 is required for SCF^TIR1/AFB^ functionality and perception of the hormone), and in *pin1* mutant (altered in auxin transport). Furthermore, NO-overproducing mutants and exogenous NO treatments decrease the expression of auxin response reporter *DR5:GUS* but promote the accumulation of PIN1 protein levels, coincident with an alteration of auxin transport. Accordingly, NO deficiency promotes an increase in the auxin maxima and a decrease in PIN1 accumulation at early developmental stages. Finally, we show that the main NO synthesis mechanism contributing to the regulation of leaf auxin maxima is related with the NOA1 pathway, since no dramatic *DR5:GUS* increase is observed in NR deficient mutants. These results confirm the relevance of NO in the distribution of auxin gradients along the leaf, mainly at early developmental stages.

## Materials and Methods

### Plant Materials and Treatments

*Arabidopsis thaliana* ecotype Columbia-0 (Col-0) is the genetic background for all wild type plants used in this work, except *cue1-1* which is in the Bensheim (Be-0) ecotype background. Seed stocks *pPIN1:PIN1-GFP*, *DR5:GUS*, and *cue1/nox1* were obtained from ABRC. Genetic crosses were performed using the *cue1/nox1* and the *noa1-1* mutant as donor in all the cases and the corresponding acceptor lines *axr1-3*, *pin1*, *pPIN1:PIN1-GFP*, and *DR5:GUS*, as described in Fernández-Marcos et al. (2011) and Sanz et al. (2014). *axr1-3*, *axr6-3*, and *pin1* mutants were a kind gift from Dr. James A.H. Murray (Cardiff University, Cardiff, United Kingdom), *nia1nia2noa1-2* mutant was a kind gift from Dr. José León (IBMCP-CSIC, Valencia, Spain), and *venosa* (*ven*) and *dov1* mutants were kindly provided by Dr. José Luis Micol (UMH, Elche, Spain). *axr1-3* mutant was generated by mutagenizing a Col-0 population with EMS (Lincoln et al., 1990), and it harbors a point mutation that changes a cysteine from the active site into an alanine, rendering the protein inactive. *Arabidopsis* plants used throughout this work were grown routinely in a growth chamber under 50–60% humidity, a temperature of 22°C, and with a 16 h light/8 h dark photoperiod at 80–100 μE m^−2^ s^−1^ in pots containing a 1:3 vermiculite:soil mixture.

For *in vitro* culture, *Arabidopsis* seeds were surface-sterilized in 75% (v/v) bleach solution (4–5% sodium hypochlorite) and 0.01% (v/v) Triton X-100 for 5 min and washed three times in sterile water before sowing. Seeds were stratified for 3 days at 4°C and then grown on MS medium (Murashige and Skoog, 1962). Petri dishes containing solid medium composed of MS basal salts and 2% (w/v) Gluc were solidified with 0.6% (w/v) agar, and the pH was adjusted to 5.7 with KOH before autoclaving. Plates were sealed and incubated horizontally in a controlled environment growth chamber. For the different treatments, seeds were sown in MS vertical sealed plates and maintain for 7, 10, 15, 21, and 30 days, after which were changed to vertical plates supplemented with 300 μM NO donor SNAP (S-Nitroso-N-acetyl-DL-penicillamine) or 1 mM NO scavenger cPTIO [2-(4-carboxyphenyl)-4,4,5,5-tetramethylimidazoline-1-oxyl-3-oxide] and incubated in the growth chamber during the different time periods.

### Measurement of Primary Root Length

Plants were grown on plates containing a modified MS medium optimized for root growth, MSR [2.3 g/L MS (Duchefa Biochemie, Haarlem, The Netherlands), 15 g/L agar], supplemented with glucose as indicated in Lechón et al. (2020). After full germination, root growth was captured by scanning plates with an Epson flatbed scanner and a resolution set to 600 ppi. Primary root length was assessed in at least three independent experiments, and individual seedlings were then measured using image analysis software Image J.

### Measurement of Phyllotactic Sequences

Phyllotactic pattern was assessed on fully grown stems of 2–3 month-old plants. The top 5 cm of the stem were not assessed because elongation was incomplete. Divergence angle and inflorescence stem length were measured simultaneously using a custom-made device similar to the one used by Peaucelle et al. (2007). The divergence angle was measured between the insertion points of two successive floral pedicels and is therefore independent of the outgrowth direction of the pedicel. Phyllotactic orientation was determined by setting the direction giving the smallest divergence angle or closest to the expected 137.5°.

### GUS Staining

GUS staining of *DR5:GUS* plants was performed using 50 mM sodium phosphate buffer (pH 7.0) containing 0.5% (v/v) Triton X-100, 1 mM K_3_/K_4_ FeCN, 0.05% (w/v) EDTA, and 1 mg/ml X-Gluc (Duchefa). At least, 10 seedlings and plants per genotype were fixed 5 min in 90% (v/v) acetone at −20°C and after this at 37°C overnight. Tissue was cleared with 70% (v/v) ethanol, and photographs were taken using a LEICA MZ 16 FA microscope coupled to a LEICA DFC 490 digital camera.

### Fluorescence Microscopy

The fluorescent photographs of seedlings and leaves grown *in vitro* and expressing the *pPIN1:PIN1-GFP* reporter gene under control of the *PIN1* promoter for confocal microscopy were mounted in 100% glycerol and taken using a LEICA TCS SP2 confocal microscope. For GFP detection, the excitation source was an argon ion laser at 488 nm and detection filters between 500 and 550 nm and for the chlorophyll channel filters between 610 and 670 nm.

### Detection of Endogenous NO

Arabidopsis seedlings between 7- and 15-day-old were incubated in a 500 μl solution containing 10 μM of 4,5-diamino-fluorescein diacetate (DAF-2DA, Sigma) in buffer 10 mM Tris-HCl, pH 7.4, during 2 h at 25°C in the dark. Seedlings were then washed three times for 15 min with fresh buffer 10 mM Tris-HCl, pH 7.4. Finally, fluorescence emitted by DAF-2DA was detected on a LEICA MZ 16 FA microscope coupled to a LEICA DFC 490 digital camera and on a LEICA TCS SP2 confocal microscope by excitation at 495 nm and emission at 515 nm. Quantification of fluorescence intensity was performed with ImageJ software. Four regions of interest (ROIs) were analyzed, corresponding to first and second leaves, shoot apical meristem (SAM), and petioles. The same area was selected in three biological replicates.

### Western Blotting

Total protein extracts for western blot analysis were obtained from leaves of *pPIN1:PIN1-GFP* transgenic and corresponding mutant lines. Tissue was placed in a standard 1.5 ml plastic microcentrifuge tube in the presence of extraction buffer (5 mM Tris-HCl pH 6.8, 2 mM EGTA, 2 mM EDTA, 2% SDS, and 1x proteases inhibitor mix, Roche) and was incubated on ice for 10 min followed by centrifugation for 10 min at 15800 g and 4°C. Protein concentration was determined by the Bio-Rad Protein Assay (Bio-Rad) based on the Bradford method (Bradford, 1976). Total protein was loaded in SDS-acrylamide/bisacrylamide gel electrophoresis using Tris-glycine-SDS buffer. Proteins were electrophoretically transferred to Inmobilon™-P PVDF membrane (Millipore) using the Trans-Blot cell (Bio-Rad). Membranes were blocked in Tris buffered saline-0.1% (v/v) Tween 20 containing 5% (w/v) blocking agent and probed with antibodies diluted in blocking buffer. Anti-GFP (Clontech), anti-Actin clone 10-B3 Purified Mouse Immunoglobulin (Sigma), ECL-Peroxidase labeled anti-mouse (Amersham), and anti-PIN1, kindly provided by Dr. Klaus Palme (University of Freiburg, Germany) antibodies were used in the western blot analyses. Detection was performed using ECL Advance Western Blotting Detection Kit (Amersham), and the chemiluminescence was detected using an Intelligent Dark-Box II, LAS-1000 scanning system (Fujifilm).

## Results

### NO Accumulates in Aerial Parts of *Arabidopsis* Seedlings

**Figure 1:**
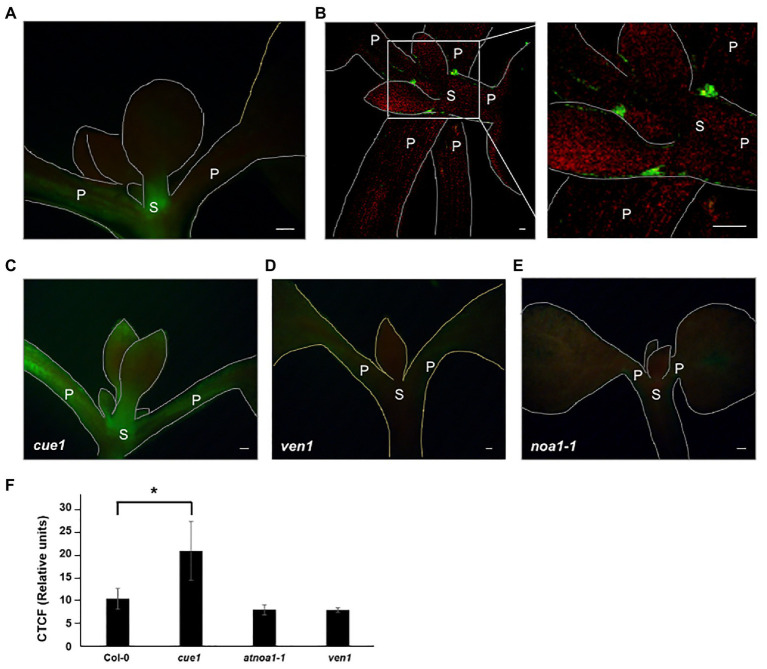
Endogenous nitric oxide (NO) levels in *Arabidopsis thaliana* young leaves. Detection of endogenous NO production using 4,5-diamino-fluorescein diacetate (DAF-2DA) in Columbia-0 (Col-0; **A,B**), *cue1/nox1* **(C)**, *ven1* **(D)**, and NO-ASSOCIATED 1 (*noa1-1*; **E**) between 7- and 15-day-old seedling leaves observed with LEICA MZ 16 FA microscope coupled to with a LEICA DFC 490 digital camera **(A,C–E)** and with a Leica TCS SP2 confocal microscope **(B)**. Quantitative data of NO-dependent DAF-2DA fluorescence leaf images. Values represent the mean ± SE (*n* = 15). Asterisk indicates statistically significant difference compared to Col-0 (*t*-test, ^*^p < 0.05; **F**). Scale bars, 50 μm. P, petiole; S, stem.

The functions of NO are closely related to the accumulation sites in different tissues along the plant. The presence of NO has been well characterized in roots, around the elongation zone (Fernández-Marcos et al., 2011) and the stem cell niche (Sanz et al., 2014), but less information is available about the aerial parts. Previous research localizes NO synthesis in primary leaves and trichomes (Tun et al., 2006; Corpas et al., 2009). Using the DAF-2DA fluorescent dye, we observed a high NO accumulation around the meristematic zones and throughout the petioles of Col-0 seedlings (Figures 1A,B).

Additionally, NO was analyzed in the NO overaccumulator *cue1/nox1*, the NO deficient *noa1-1* and *venosa1* (*ven1*) mutants in seedlings between 7- and 15-day-old, using Col-0 as the wild type (Figures 1C–F). In the wild type fluorescence was mainly observed along the meristematic zones (Figure 1A), as previously described (Corpas et al., 2009). The greater NO accumulation corresponded to younger leaves of *cue1/nox1* as expected, while *ven1* and *noa1-1* mutants showed a decrease along the aerial part (Figures 1C–F). These results revealed that NO production was specifically detected in the first leaves formed, where a physiological effect is presumed, since DAF-2DA has the advantage to detect NO accumulation very close to the production sites in cells.

### NO Homeostasis Mutants Present Abnormal Leaf Phenotypes

**Figure 2:**
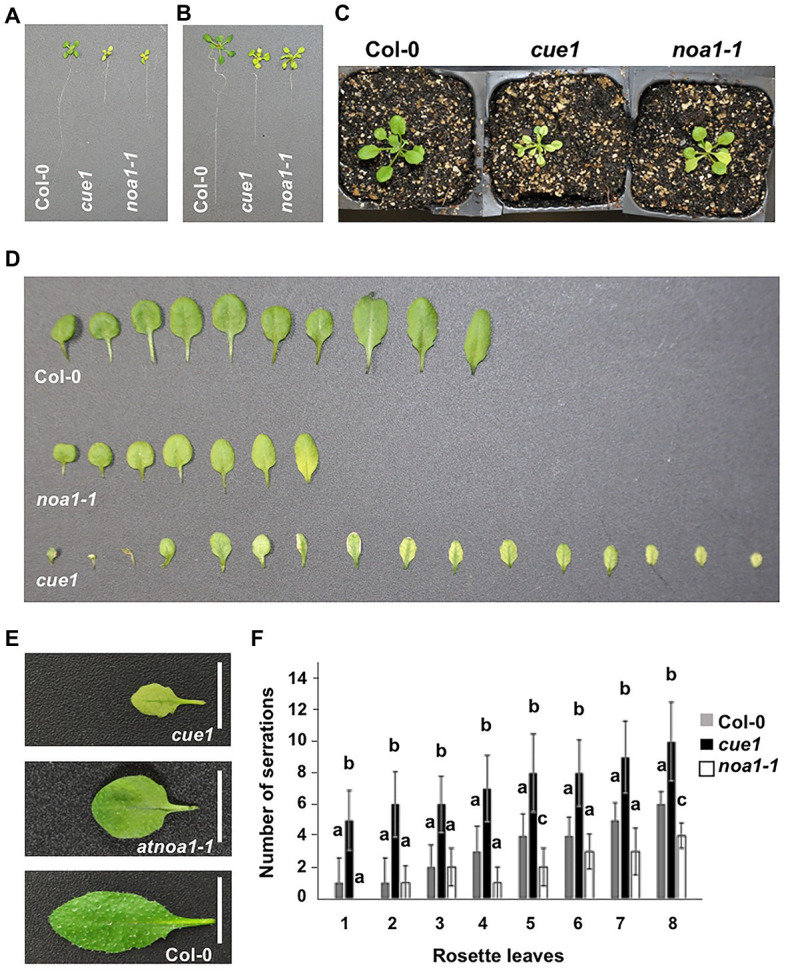
Leaf phenotype of NO homeostasis mutants. Phenotype of 15-day-old seedlings **(A)**, 21-day-old seedlings **(B)**, 21-day-old plant rosettes **(C)**, and 30-day-old plant leaves **(D,E)**. Number of serrations in 30-day-old *Arabidopsis* leaves **(F)**. The developmental number of the leaf is represented on the *X*-axis and cotyledons are not included. Values represent the mean ± SD (*n* = 10). Col-0, the ecotype used as a control is compared with the NO overproducer *cue1/nox1* mutant and the NO deficient *noa1-1*.

Nitric oxide was described to be involved in the regulation of root development (Stöhr and Stremlau, 2006; Fernández-Marcos et al., 2011, 2012; Bai et al., 2014) and root stem cell maintenance (Sanz et al., 2014), but more information is needed to clarify its role during leaf morphology. A visual analysis of NO homeostasis mutants showed apparent defects associated with leaf morphology (Figures 2A–E) and structural characteristics, like the number of serrations (Figure 2F). The reduced number of true leaves in *noa1-1* mutant showed a pale phenotype, with premature senescence in the oldest ones, as previously reported (Liu and Guo, 2013; Lv et al., 2019), and a low number of serrations. On the opposite, the NO overaccumulator *cue1/nox1* exhibited a high number of smaller leaves, with a remarkable *venosa* phenotype, characterized by a different pigmentation between the green veins and the pale interveinal regions (also known as *reticulate*; Berná et al., 1999; Robles and Micol, 2001), and a greater number of serrations (Figure 2F).

A highly similar pale phenotype was also described as a consequence of mutations in the *RETICULATA-RELATED* gene family (González-Bayón et al., 2006; Pérez-Pérez et al., 2013) and *DIFFERENTIAL DEVELOPMENT OF VASCULAR ASSOCIATED CELLS 1 (DOV1)* gene (Kinsman and Pyke, 1998; Rosar et al., 2012), independently of NO levels. Analyzing the root length variation in response to exogenous NO treatment, only *cue1/nox1* mutant underwent a significant reduction, as previously described (He et al., 2004; Fernández-Marcos et al., 2011), while we did not found any common correlation between the rest of *venosa* mutants, compared to the wild type (Supplementary Figure 1).

### NO Mutants Exhibit Additional Growth Defects During Aerial Part Development

**Figure 3:**
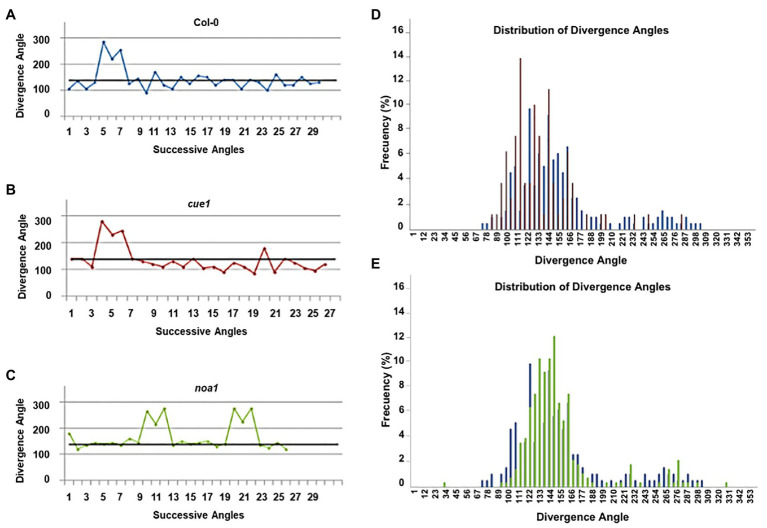
Phyllotactic patterns of NO homeostasis mutants. Representative angle sequences of Col-0 **(A)**, *cue1* **(B)**, and *noa1* **(C)**. The black lines represent the canonical angle sequence (137.5°) and colors in the graph correspond to Col-0 (blue), *cue1* (red), and *noa1* (green). Distribution of divergence angles for all analyzed phyllotactic sequences of Col-0 vs. *cue1* **(D)** and Col-0 vs. *noa1* **(E)**. At least, 10 plants per genotype were analyzed.

The phyllotaxis of a plant is defined as the stereotyped spatiotemporal pattern of organ initiation that results in the recognizable architecture of the plant. In the shoot apex, phyllotaxis is driven by the pattern of auxin distribution (Reinhardt et al., 2003). Given that NO homeostasis mutants present abnormal leaf phenotypes, we also studied organ phyllotaxis in *cue1* and *noa1* mutants to find out whether altered NO levels were translated into a phyllotactic phenotype (Figures 3A–D). For this reason, plants were grown for 2–3 months until their inflorescences were fully elongated, and the position of the plants was randomized to minimize environmental fluctuations. To assess the phyllotactic pattern, we measured the length of the primary inflorescence and the divergence angle between siliques. *Arabidopsis thaliana* has a spiral phyllotaxis, with a divergence angle of 137.5° between successive organs, which is usually termed *α* or canonical angle (Galvan-Ampudia et al., 2016). However, a notable amount (83%) of wild type plants also display organ permutations along the stem, that are measured as an angle sequence of 2α, 360 - α, and 2α and give an “M motif” when a phyllotactic sequence is represented in a graph (Figure 3A). These permutations are the result of two organs initiated simultaneously in the SAM, and lead to a normal distribution or permutation depending on which organ is positioned above the other after internode elongation (Galvan-Ampudia et al., 2016). The phyllotactic sequences were quite similar for all three genotypes, as the relative angle between siliques and the position of their initiation relative to the center of the SAM were unaffected in *cue1* and *noa1* (Figures 3B,C). When we analyzed the frequency of divergence angles for all the phyllotactic sequences analyzed, most siliques were separated by angles that follow a normal distribution around the canonical angle (Figure 3D).

We also found that the number of permutations might be related to the endogenous NO content of the plant, since all *noa1* plants presented permutations, 17% more than wild type plants. On the other hand, only 67% of *cue1* plants had permutated siliques (Supplementary Table 1). The number of successive permutations, that is, of more than two siliques involved in successive permutations, was higher for *noa1* than for Col-0 and, especially, *cue1* plants. However, the permutation frequency for both mutants (one and three permutations per plant for *cue1* and *noa1*, respectively) is within the usual range of permutations in Col-0 plants (Besnard et al., 2014).

In addition to phyllotactic pattern analysis, we compared the length of the primary inflorescences of Col-0, *cue1*, and *noa1*. While the inflorescence of *cue1* is indeed 61% shorter than that of Col-0, both mutants have smaller inflorescences, as the inflorescence of *noa1* is also 26% shorter. In the same way, both mutants have a higher silique production rate than wild type plants, as *cue1* has 2.92 ± 0.19 siliques/cm of inflorescence, 48% more than Col-0, which only has 1.53 ± 0.12 siliques/cm, and *noa1* has 2.36 ± 0.12 siliques/cm, 35% more siliques than the wild type. However, the number of siliques per plant is quite different among the genotypes, as *noa1* has 13% more siliques per plant compared to the wild type, and *cue1* has 24% less siliques per plant. Therefore, the increase in silique production seems to be a function of the decrease in inflorescence length and not a decrease in the rate of organ production, the plastochron, which, according to this data, seems to be unaltered in both NO homeostasis mutants (Supplementary Table 1). Recently, Ware et al. (2020) have proposed a framework to explain the arrest of *Arabidopsis* inflorescence as a local process driven by auxin export from fruit proximal to the inflorescence apex.

### Auxin Transport and Responses are Altered in *cue1*

**Figure 4:**
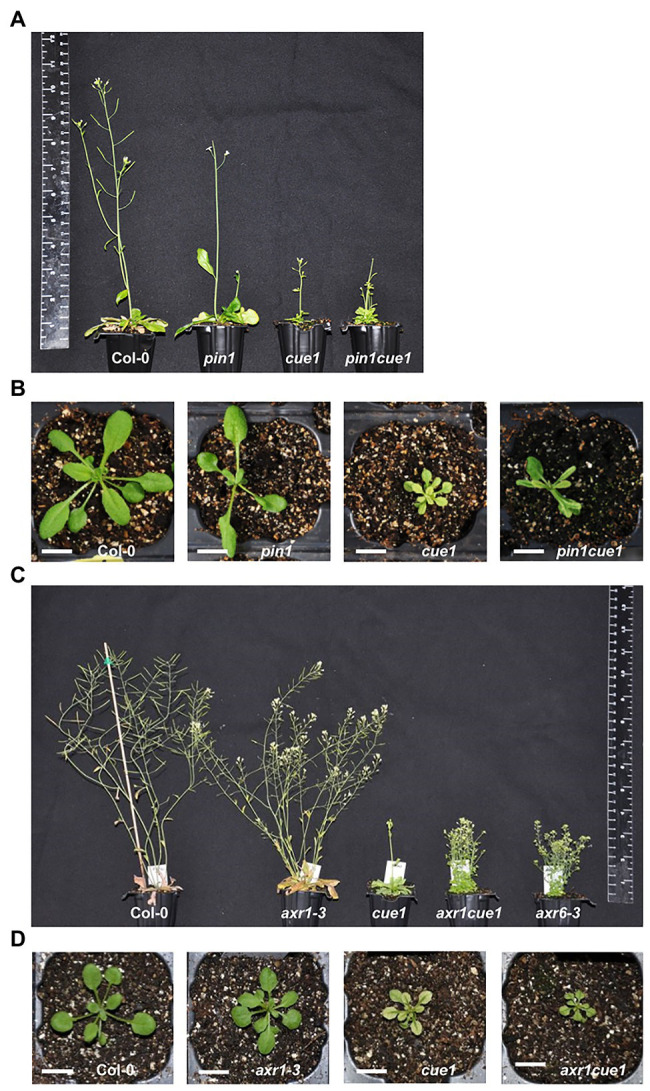
The *cue1* mutation alters auxin transport and increases auxin response impairment. Phenotype of 30-day-old plant rosettes **(A)** and 21-day-old plant rosettes **(B)** in wild type (Col-0), *pin1* mutant altered in auxin transport, NO overproducer *cue1* mutant, and *pin1cue1* double mutant. Phenotype of 50-day-old plant rosettes **(C)** and 21-day-old plant rosettes **(D)** in wild type (Col-0), *axr6-3*, *axr1-3*, *cue1*, and double *axr1-3cue1*. Plants were grown on MS for 14 days at 20°C, then transferred to soil and grown in the greenhouse.

In Fernández Marcos et al. (2011), we showed that high levels of NO reduce the acropetal auxin transport. Furthermore, we observed certain similarities between the phenotypes of the *cue1* and *pin1* mutants. The loss of function of PIN1 protein severely affects the formation of plant organs. Thus, the phenotype of an adult *pin1* plant is characterized by the presence of leaves with altered morphology that lack serrations and a bare inflorescence stem that does not produce flowers or, if it does, they are sterile. This line was crossed with the *cue1* mutant to obtain a *pin1cue1* homozygous double mutant that gave rise to additive phenotypes with the appearance of the characteristic *venosa* phenotype and a bare inflorescence in adult plants (Figures 4A,B). Interestingly, the root length of the double *pin1cue1* mutant is on average shorter than that of the parentals *cue1* and *pin1*. These differences in average suggest that the inhibition of root growth associated with *cue1* entails the existence of additional mechanisms to the possible *pin1* loss-of-function (Supplementary Figure 2A).

The expression of synthetic auxin response reporter construct *DR5:GFP* was decreased in the root of the *cue1* mutant, confirming that NO accumulation also inhibits auxin signaling in the root apical meristem (RAM; Fernández-Marcos et al., 2011). In order to establish the extent of its auxin signaling impairment and relation to the functionality and perception of the hormone, we introduced the *cue1* mutation into the loss-of-function *axr1* (*axr1-3*) line (Lincoln et al., 1990) and studied the phenotype of the *axr1cue1*. The resulting double mutant mimicked to some extent, the adult plant phenotype displayed by the *axr6* mutant (*axr6-3*; Quint et al., 2005; Figures 4C,D).

*AUXIN RESISTANT 1* encodes a RUB-conjugating enzyme which regulates auxin Skp1/Cullin/F-box (SCF) activity (del Pozo et al., 1998), while *AXR6* encodes the CUL1 subunit of the SCF complex (Hellmann et al., 2003). Loss-of-function *axr1* and *axr6* plants exhibited growth defects, including a slight reduction in plant height and an increased number of lateral branches. The adult phenotype of *axr1-3* is significantly less severe than that of *axr6-3* (Lincoln et al., 1990; Quint et al., 2005). Seven-week-old *axr1cue1* plants display reduced stature, an increased number of branches compared to *axr1* plants, and epinastic rosette leaves, similar to *axr6* plants (Figure 4C). These phenotypes suggest that introducing *cue1* in *axr1* increases its resistance to auxin. Contrary to the phenotype exhibited in the above-ground organs, examination of the primary root length of 7-day-old *axr1cue1* (1.34 ± 0.22 cm), *axr1* (1.68 ± 0.28 cm), *cue1* (1.20 ± 0.17 cm), and wild type seedlings (1.54 ± 0.24 cm) revealed that introducing the *axr1* mutation into the *cue1* background had no effect (*p* > 0.05) in root growth inhibition (Supplementary Figure 2B), highlighting specific NO effects depending on plant tissue.

### NO Disturbs the Accumulation of Auxin Maxima

Reported evidences support that NO modulates root architecture by the control of auxin spatial pattern (Fernández-Marcos et al., 2011), but it remains unclear whether the crosstalk with NO could determine leaf phenotype. Leaf development is influenced by spatial patterns of auxin response based on auxin gradients (Avsian-Kretchmer et al., 2002; Heisler et al., 2005; Cheng et al., 2007). Using the auxin output reporter *DR5:GUS* (Ulmasov et al., 1997) for canonical auxin signaling, response-auxin sites were visualized in the leaf elongation tips during primordium development, that progressively moved to the margins by basipetal transport, hydathodes, trichomes, and mesophyll cells (Aloni et al., 2003).

**Figure 5:**
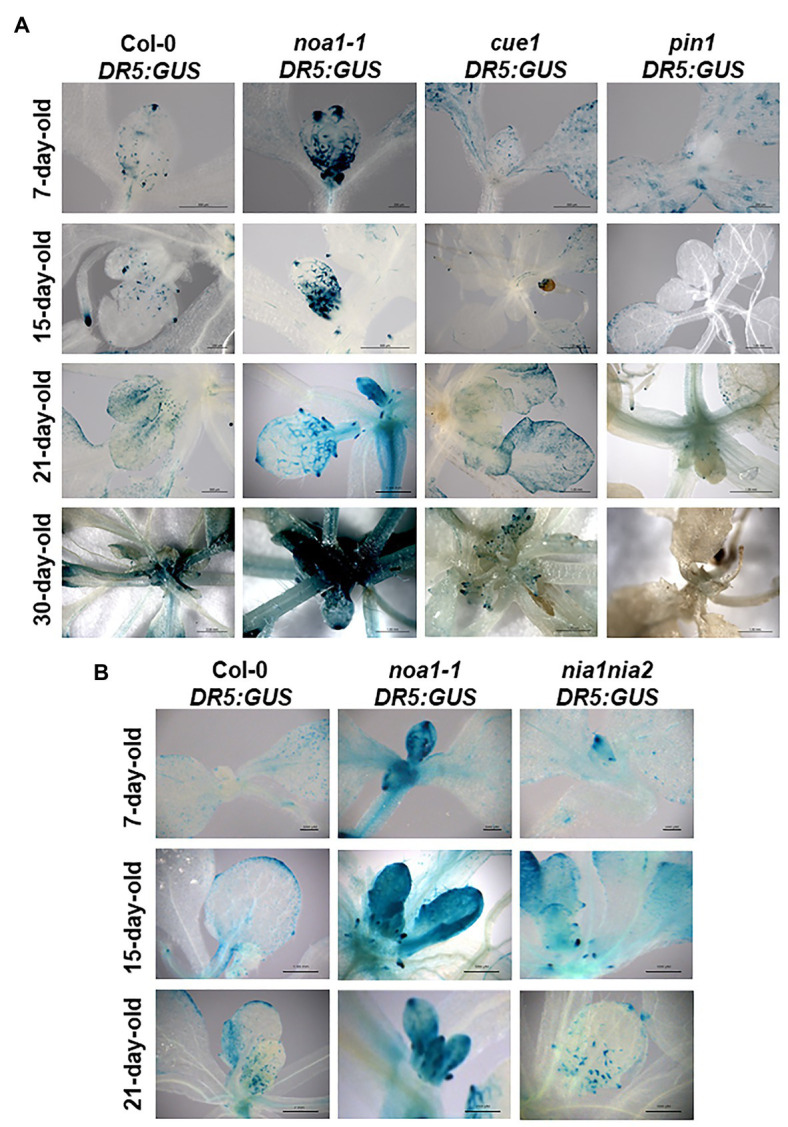
Auxin response is impacted by mutations in *NOA1* and *CUE1*. Pattern of *DR5:GUS* expression in transformed *A. thaliana* Col-0 and *noa1-1*, *cue1-1*, and *pin1* mutant lines, showing histochemical localization of ß-GUS activity during morphogenesis in first and second leaves of 7-day-old seedlings, sixth and seventh leaves of 15-day-old seedlings, leaves of 21-day-old plants, and 30-day-old plants **(A)**. Scale bars, between 200 and 1,000 μm. Histochemical localization was also showed in *nia1nia2* NO deficient mutant in 7-, 15-, and 21-day-old plants **(B)**. At least, 10 seedlings and plants per genotype were analyzed all showing a similar expression pattern and a representative image was selected.

We observed the expression pattern of *DR5:GUS* under wild type and different NO mutant backgrounds (Figure 5, Supplementary Figure 3) and subjected to NO treatments (Supplementary Figure 4), in order to analyze the effect of NO in the auxin maxima distribution.

In this context, NO overproducer mutants (Figure 5A, Supplementary Figure 3) and exogenous NO treatments (Supplementary Figure 4) decreased the expression of the auxin response reporter *DR5:GUS*, in contrast to the NO deficient *noa1-1* (Figure 5, Supplementary Figure 3) and NO scavenging (Supplementary Figure 4), which enhanced the detection of auxin maxima. Additionally, the pattern of auxin maxima in *cue1/nox1* mutant was restored after 48 h of NO scavenger treatment (Supplementary Figure 4). These results were consistently observed during different developmental stages of leaf primordium (i.e., from the first and second leaves to the sixth true leaf stage), avoiding the highly pleiotropic phenotypes of NO homeostasis mutants compromise synchronization of growth to compare to the wild type.

In order to test the contribution of the two main pathways involved in NO production into the regulation of the pattern of auxin maxima, we used the available genetic tools for *NIA1*, *NIA2*, and *NOA1*. The most significant changes in the *DR5:GUS* expression pattern compared to the wild type were observed in the *noa1-1* deficient mutant (Figure 5B), while *nia1nia2* expression pattern was close to the wild type. These results suggest that NOA1 could have a main role in NO production during the regulation of auxin maxima distribution in shoots.

### NO Promotes PIN-FORMED1 Accumulation in Aerial Parts

**Figure 6:**
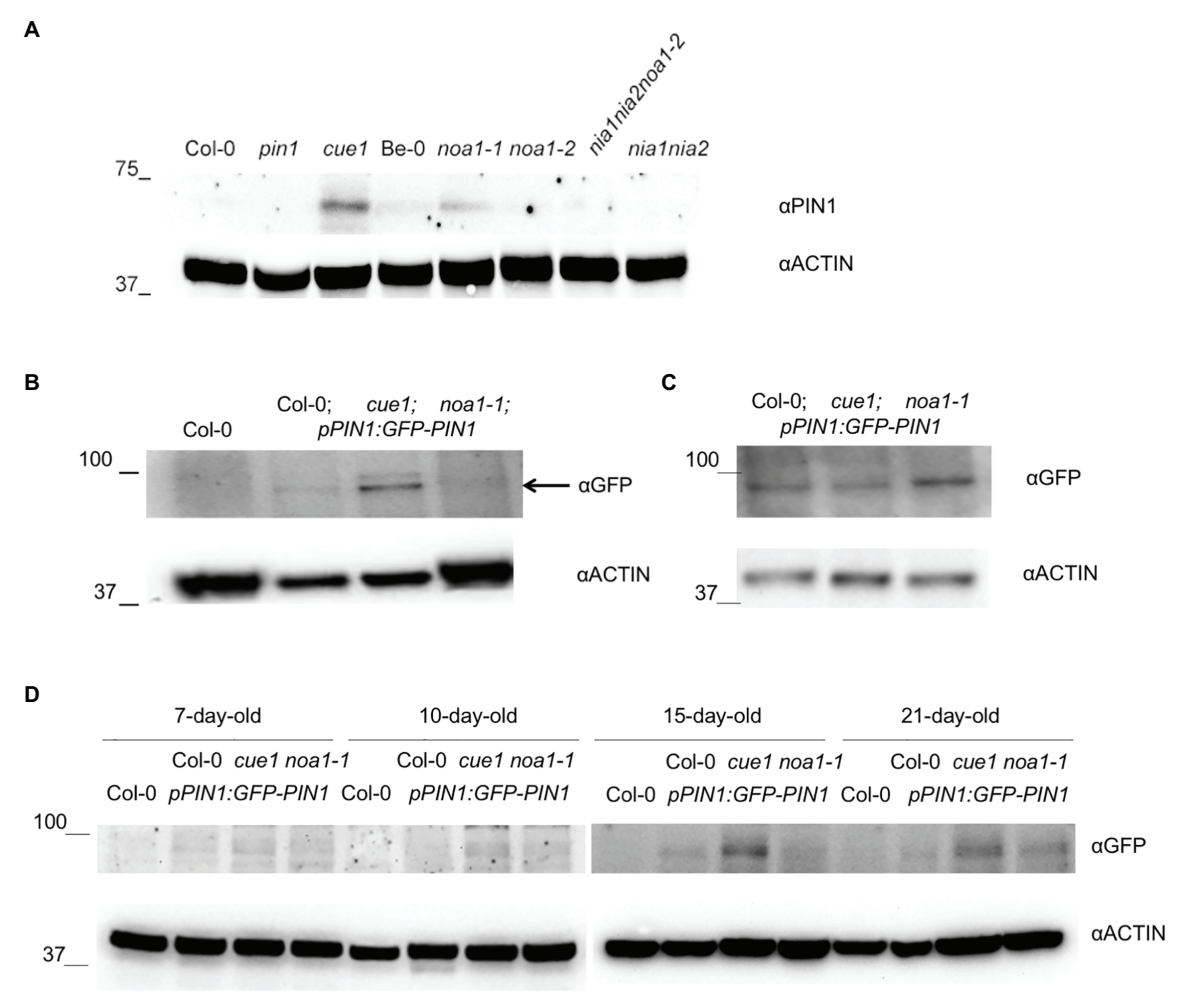
Effect of NO homeostasis mutants on PIN1 protein accumulation. Immunoblot analysis with anti-PIN1 in 30-day-old, *A. thaliana* rosette leaf extracts grown on soil from Col-0, *pin1*, *cue1/nox1*, *noa1-1*, *noa1-2*, *nia1nia2noa1-2*, and *nia1nia2* mutants **(A)**. Immunoblot analysis with anti-GFP in 21-day-old leaf extracts from Col-0 and *pPIN1:PIN1-GFP* transgenic lines in Col-0, *cue1/nox1*, and *noa1-1* backgrounds **(B)**. Black arrow indicates PIN1-GFP quimeric protein detection. **(C)** Immunoblot analyses with anti-GFP antibody in 15-day-old *Arabidopsis* root extracts from *pPIN1:PIN1-GFP* transgenic lines in Col-0, *cue1/nox1*, and *noa1-1* backgrounds grown on MSR. Actin protein levels were also determined as loading control. **(D)** Immunoblot analysis with anti-GFP in Col-0 and *pPIN1:PIN1-GFP* transgenic lines in Col-0, *cue1/nox1*, and *noa1-1* backgrounds using 7-, 10-, 15-, and 21-day-old rosette leaf extracts grown *in vitro*. Actin protein levels were also determined as loading control.

Auxin responses are based on dynamic spatial patterns mediated by the efflux transporter proteins, PIN-FORMED family, whose asymmetric localization promotes changes in polar auxin movements, leading to the correct hormone distribution (Palme and Gälweiler, 1999; reviewed in Friml, 2003). Within this family, PIN1 polar localization presents a prominent role determining auxin distribution in the aerial part (Benková et al., 2003). Previously, Fernández-Marcos et al. (2011) showed a PIN1 decrease in primary roots under NO treatments and in the *cue1/nox1* NO overproducer mutant. Additionally, the root meristem size of this mutant resembles that of the *pin1* knock-out mutant phenotype (Fernández-Marcos et al., 2011). To determine whether NO can affect PIN1 accumulation pattern during leaf development, we analyzed PIN1 protein levels in *Arabidopsis* young and rosettes leaves in different NO homeostasis mutant backgrounds (Figure 6).

Interestingly, we found a higher PIN1 accumulation in *cue1/nox1* mutant compared to the wild type Col-0, along the different developmental stages of the aerial part analyzed (i.e., 7-, 10-, 15-, and 21-day-old seedlings grown *in vitro* and 21- and 30-day-old plant rosettes grown on soil; Figure 6). Oppositely, a decrease of PIN1 accumulation is observed in all the NO deficient mutants analyzed (*noa1*, *nia1nia2*, and *nia1nia2noa1-2*; Figures 6A,B,D), except in 30-day-old *noa1-1* plant rosettes grown on soil.

**Figure 7:**
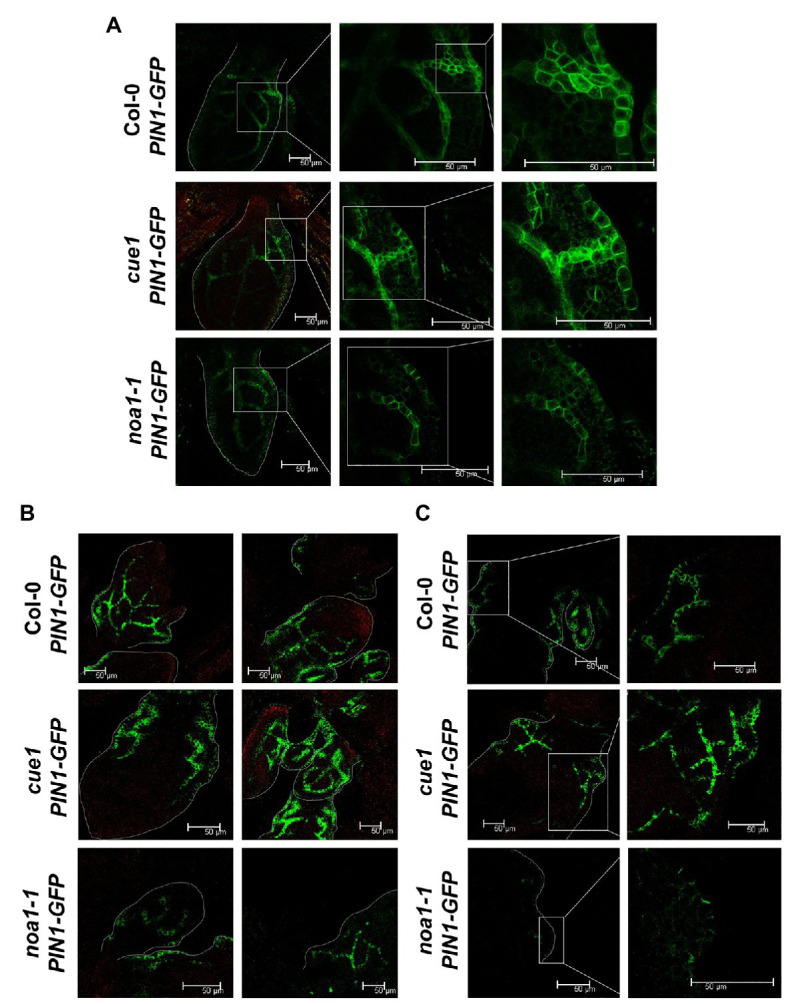
Distribution of PIN1 in aerial parts of NO homeostasis mutants. Confocal micrographs showing the expression of *pPIN1:PIN1-GFP* in *A. thaliana* grown *in vitro* seedlings first or second leaf margin **(A)**, in sixth and seventh leaf margin **(B)**, and in eleventh rosette leaf margin **(C)**. Col-0; *pPIN1:PIN1-GFP, cue1; pPIN1:PIN1-GFP* and *noa1-1;* and *pPIN1:PIN1-GFP* lines are shown. Scale bars, 50 μm.

To better understand the role of NO in PIN1 localization and distribution during early development of leaves, we carried out a confocal analysis showing the distribution of PIN1-GFP protein in young leaves of seedlings grown *in vitro* and expressing the *pPIN1:PIN1-GFP* reporter gene under control of the *PIN1* promoter (Figure 7). The different NO homeostasis mutant backgrounds analyzed included the NO overproducer *cue1/nox1* and the NO deficient *noa1-1* mutants.

Thus, the quantitative results obtained with PIN1 accumulation (Figure 6) are fully supported by the confocal analysis, where we observed a reduction in the PIN1-GFP fluorescence pattern along the venations and the leaf margins in the *noa1-1* mutant, in comparison to the wild type and *cue1/nox1* background (Figure 7). This pattern was conserved in *noa1-1;pPIN1:PIN1-GFP* seedlings at every developmental stage analyzed from the first or second leaf margin (Figure 7A), in sixth and seventh leaf margin (Figure 7B) and in eleventh rosette leaf margin (Figure 7C).

All together, these results suggest an opposite effect for NO during the regulation of PIN1 distribution in aerial parts compared to the previously reported in roots (Fernández-Marcos et al., 2011; Figure 6C). PIN1 trafficking is controlled in part by auxin (Paciorek et al., 2005), and free-auxin production shows a differential pattern in shoots compared to the root tips (Aloni et al., 2003). This is not surprising, because leaf and root primordia initiation and development are differentially regulated (Elliott et al., 1996; Malamy and Benfey, 1997).

## Discussion

Plants have evolved to adapt in a changing environment. Leaves exemplify this plasticity because of their highly variable morphology. Leaf organs play a key role in light capture and carbon fixing, modulating their structure depending on the external and internal cues. The complex network controlling leaf phenotype is composed by the interplay between hormones, transcription factors, and other components, such as secondary messengers or key gasotransmitters, like NO (Albertos et al., 2016) or oxygen. In roots, NO participates in many developmental cues (Pagnussat et al., 2003, 2004; Correa-Aragunde et al., 2006; Méndez-Bravo et al., 2010; Fernández-Marcos et al., 2011, 2012), but little is known about the regulation of leaf structure. Different NO homeostasis mutants present severe defects related to leaf phenotype, such as leaf number and margin, serrations, phyllotactic pattern, and number of siliques (Figures 2, 3, Supplementary Table 1). Based on our previous results that link NO effect to auxin transport and signaling inhibition in roots (Fernández-Marcos et al., 2011), we crossed the NO overproducer mutant *cue1* with the loss-of-function mutants *pin1* and *axr1*, in order to analyze the resulting phenotype (Figure 4). Double mutant *pin1cue1* shows an additive phenotype (Figures 4A,B), while *axr1cue1* resembles the partial auxin insensitivity displayed by *axr6* (Quint et al., 2005) and shows defects, such as reduced stature, higher number of branches, and epinastic rosette leaves (Figures 4C,D). Root length of the double *pin1cue1* mutant was shorter than the corresponding parentals (Supplementary Figure 2A), suggesting the existence of additional mechanisms during the inhibition of root growth. Interestingly, no inhibition effect was observed during root growth of *axr1cue1* (Supplementary Figure 2B), highlighting a great tissue specificity.

Expression of a NO degrading dioxygenase (NOD) has been described to initiate leaf senescence (Mishina et al., 2007). This result is in agreement with our observations of premature leaf senescence in 30-day-old plant of the NO deficient *noa1-1* mutant (Guo et al., 2003; Figure 2). Additionally, a delay effect in leaf senescence induced by methyl jasmonate was observed when low salicylic acid concentrations are used, which depends on NOA1 pathway for NO production (Ji et al., 2016). Green reticulation on a paler lamina phenotype is a common feature between *venosa* and NO overaccumulator *cue1/nox1* mutants (Li et al., 1995). Nevertheless, the analysis of primary root length (Supplementary Figure 1) showed that not all the lines behave in a similar way to the NO donor application, suggesting that *ven* phenotype is independent of NO levels. The rescue of *noa1* pale phenotype by NO exogenous treatment was previously described (Guo et al., 2003), but its specific role at this level remains unclear. This result highlights the complex network involved in leaf morphology and structure, where NO emerges as an important player.

Sites of NO synthesis have been described in primary leaves and trichomes (Tun et al., 2006; Corpas et al., 2009). Using the DAF-2DA fluorescence probe, we observed endogenous NO accumulation in younger seedling leaves, mainly in the SAM (Figure 1). As expected, the observed pattern was increased in the NO overaccumulator *cue1*/*nox1* mutant background (Figure 1). Endogenous NO detection was also observed in the RAM (Fernández-Marcos et al., 2011; Sanz et al., 2014), suggesting a potential role for this molecule inside these specific tissues.

Auxin distribution regulates organ formation and sites of maxima accumulation point to the primordium initiation (Aloni et al., 2003; Benková et al., 2003). Using the auxin response reporter *DR5:GUS* (Ulmasov et al., 1997), we analyzed the NO effect in the sites of high free-auxin distribution of *Arabidopsis* seedling leaves. Auxin maxima were observed mainly in the leaf tip, that progressively moved to the margins, hydathodes, and basal sites (Figure 5), where venation formation occurs, as was previously described (Aloni et al., 2003; Mattsson et al., 2003). Lower NO levels increase auxin accumulation along the younger leaves and during whole plant development, finding the opposite effect under higher NO levels (Figure 5, Supplementary Figures 3, 4). To deep further into the source of NO production in this process, we detected the strongest ß-GUS activity in *noa1-1* mutant, suggesting that NOA1 is the main enzymatic mechanism involved (Figure 5). Extensive crosstalk between NO and auxin has been reported at the level of synthesis, transport, perception (i.e., *S*-nitrosation of the auxin receptor protein TIR1), and signaling (i.e., degradation of Aux/IAAs and consequent activation of *AUXIN RESPONSE FACTOR* (*ARF*) genes; reviewed in Sanz et al., 2015).

Auxin spatial and temporal pattern involves polar transport mediated mainly by the PIN1 efflux carrier (Benková et al., 2003). In order to decipher the mechanisms by which NO modifies auxin maxima distribution, we checked PIN1 localization and accumulation in *Arabidopsis* leaf primordium and seedling leaves by confocal microscopy and western blot. Using the *pPIN1:PIN1-GFP* construction, we visualized PIN1 along the leaf margins and developing vasculature, as was previously localized (Benková et al., 2003; Reinhardt et al., 2003; Heisler et al., 2005; Hay et al., 2006). Interestingly, in *cue1/nox1* mutant, we could detect an increase in PIN1 accumulation by western blot opposite to the effect of NO on PIN stability reported in roots (Figure 6), but the distribution pattern remains unchanged at the developmental stages analyzed (Figure 7). In contrast, *noa1-1* mutant showed a dramatic decrease in PIN1-GFP fluorescence. Previous research showed that PIN1 activity promotes the formation of leaf serrations (Hay et al., 2006) and *cue1/nox1* present leaf margins with more serrations compared to the Col-0 wild type (Figure 2), suggesting that NO could modify leaf morphology through PIN1 regulation. Furthermore, Fernández-Marcos et al. (2011) showed a decrease in PIN1 along the root meristem in the presence of NO, evidencing a differential effect of NO in the auxin maxima accumulation between shoots and roots. Supporting this result, Otvös et al. (2005) found that promotion of cell division mediated by auxin in cell derived from alfalfa leaf protoplasts, was increased in the presence of NO, whereas in roots, the opposite effect was observed (Fernández-Marcos et al., 2011).

PIN-FORMED 1 polar localization is regulated by several factors. Among them, stand out key proteins such as the transcriptional activator MONOPTEROS (MP), which belongs to the ARF family (Wenzel et al., 2007; Schuetz et al., 2008), GNOM ARF GEF affecting the endocytosis recycling (Steinmann et al., 1999; Geldner et al., 2003) and the protein kinase PINOID, that modulates PIN1 apical-basal location (Friml et al., 2004). Two feedback loops are described for PIN1 polar location and auxin maxima distribution along the leaf margins. The first links auxin transport to its own distribution through PIN1 localization. In the second loop, CUP-SHAPED COTYLEDON 2 (CUC2) controls PIN1 reorientation, being repressed by auxin at the same time (Bilsborough et al., 2011). To get further insights into the mechanisms by which NO could control PIN1 localization and the pattern of auxin maxima, we searched for *in silico* putative targets of *S*-nitrosation, in the main players involved in this process, by using the GPS-SNO 1.0 software (Xue et al., 2010). Interestingly, PINOID, CUC2, MP, and GNOM ARF GEF present Cys residues susceptible to suffer this post-translational modification (Table 1). Remarkably, MP and GNOM ARF GEF, with three and four SNO hypothetical modifications, respectively, suggest that NO can modulate auxin transport at different levels including transcriptional regulation and endocytosis recycling. Indeed, the PIN1 endocytic recycling cycle depends on the actin cytoskeleton (Geldner et al., 2001), whose structure is in part controlled by *S*-nitrosation (Lindermayr et al., 2005; Rodriguez-Serrano et al., 2014).

**Table 1:** Analysis of putative *S*-nitrosation modifications in Cys residues from the main proteins implicated in the regulation of PIN-FORMED 1 (PIN1) by using the GPS-SNO 1.0 software.

PROTEIN	PROCESS	TOTAL CYS NUMBER	PUTATIVE SNO CYS
PINOID	Apical-basal localization	5	1
ARF GEF GNOM	Endocytosis recycling	32	4
MONOPTEROS	Transcriptional activator	19	3
CUP SHAPED COTYLEDON 2	Reorientation in response to auxin	6	1

Together, these results provide new evidences on the regulation of NO during leaf morphology, as opposed to previous research on root development, and suggesting the tissue specificity for NO function. NO homeostasis mutants are impaired in different parameters related to aerial part, highlighting leaf shape, serrations, and premature senescence. Auxin maxima decrease can be linked to PIN1 greater accumulation, as a compensatory mechanism, in order to maintain the auxin spatial pattern along leaf margins and venations. Leaf development is tightly modulated by external and internal cues, coupling shape with functionality to maximize plant adaptation to the environmental changes. Results presented in this work suggest an extensive crosstalk between different signals (i.e., auxin and NO) and provide putative NO targets to elucidate the complete network during shoot development.

## Data Availability Statement

The original contributions presented in the study are included in the article/Supplementary Material; further inquiries can be directed to the corresponding author.

## Author Contributions

IS-V and TL performed research. MF-M and LS generated transgenic lines and performed root growth assays. IS-V and OL analyzed data and wrote the paper. OL conceived and designed research and supervised the work. All authors discussed the results and commented on the manuscript. All authors contributed to the article and approved the submitted version.

### Conflict of Interest

The authors declare that the research was conducted in the absence of any commercial or financial relationships that could be construed as a potential conflict of interest.
