# Supplementary material for: Nitric Oxide Alters the Pattern of Auxin Maxima and PIN-FORMED1 During Shoot Development
Source: Front Plant Sci. 2021 Apr 26;12:630792. doi: 10.3389/fpls.2021.630792 (PMC8189175; doi:10.3389/fpls.2021.630792)
Supplement: Supplementary file 1 [file Data_Sheet_1.PDF]

Supplementary material

Supplementary Table 1. Quantification of permutations in the phyllotactic sequences, inflorescence stem elongation, length of the primary inflorescences, number of siliques per plant and silique production rate (siliques/cm of inflorescence) in Col-0, *cue1*, and *noa1,* was assessed as explained in the Material and methods section. At least 10 plants per genotype were analyzed and values represent the mean ± SE.

Arabidopsis line	Col-0	*cue1*	*noa1*
Phyllotactic sequences	12	3	7
Permutations	25	2	20
Succesive permutations	2	-	3
Plants without permutations	2 (17%)	1 (33%)	0
Permutations per plant	2	1	3
Inflorescence stem lenght (cm)	23.7±1.3	9.3±0.3	17.6±1.5
Number of siliques per plant	36±2.7	27.3±2.6	41.6±3.7
Silique production (siliques/cm)	1.53±0.12	2.92±0.19	2.36±0.12

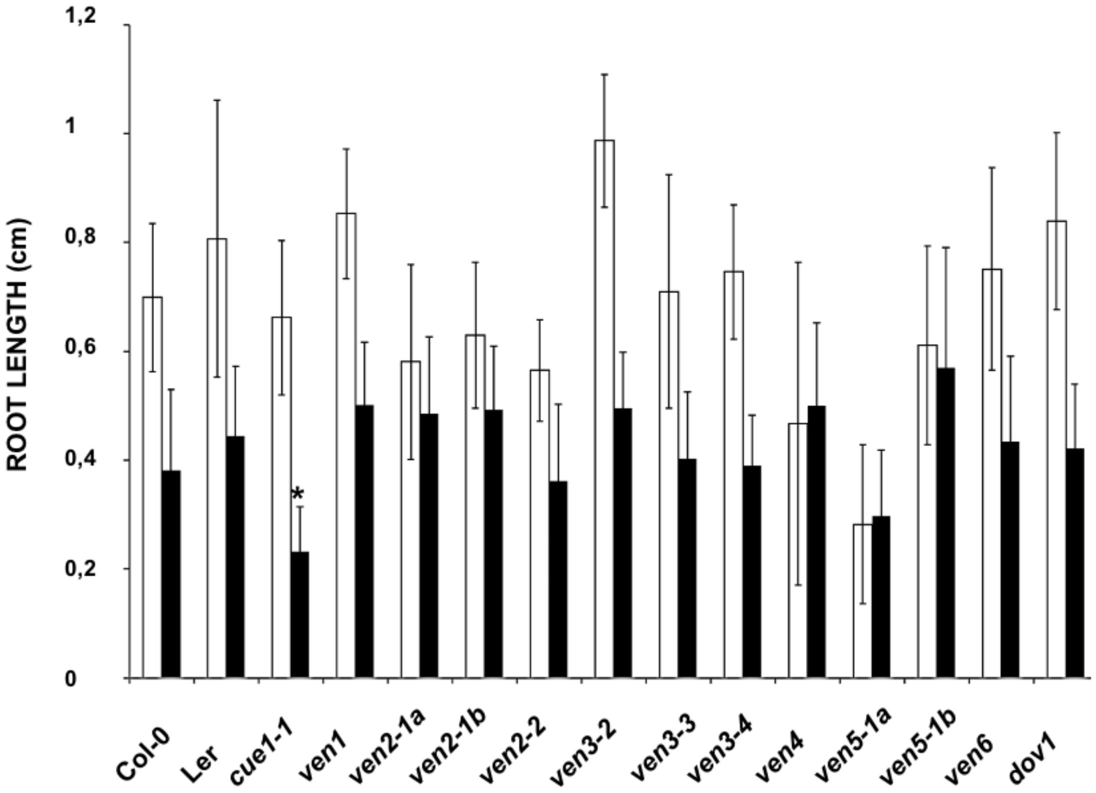

Supplementary Figure 1. Root length analysis of 7-day-old seedlings of Col-0, L*er*, NO overaccumulator mutant *cue1-1*, *ven* and *dov1* mutans under NO treatment and control conditions in horizontal plates. White bars correspond to MS media and black bars to MS supplemented with the NO donor SNAP (300μM). Means ± SE of three biological replicates are represented (n = 25). Asterisk indicates a statistically significant difference compared to Col-0 (*t*-test, $^{*}P$<0,05).

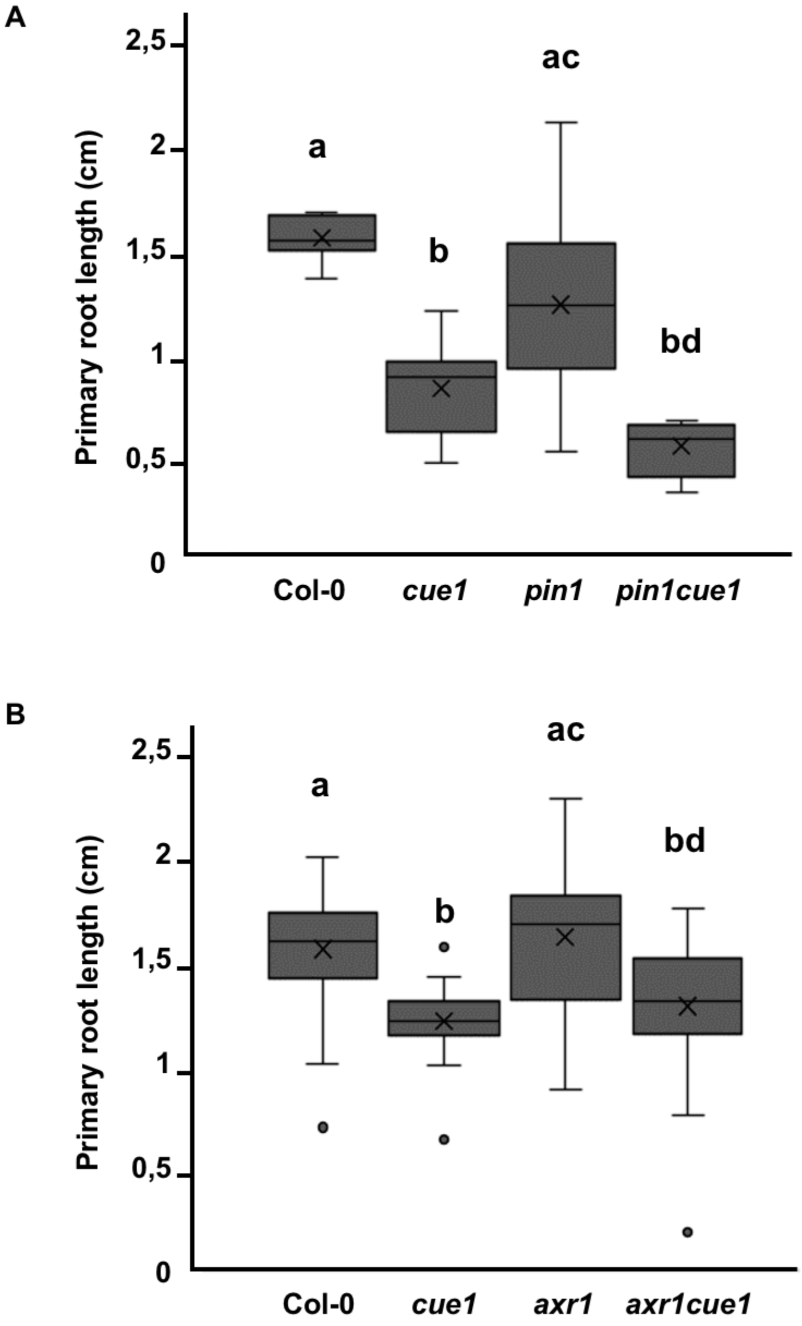

Supplementary Figure 2. Primary root elongation in Col-0, NO overproducing mutant *cue1, pin1* auxin transport altered mutant and the corresponding *pin1cue1* double mutant (A), *axr1* auxin mutant and the corresponding *axr1cue1* double mutant (B). Root length of 7-day-old seedlings grown vertically on MSR (n = 32), as explained in the Material and methods section. The diagrams show data between the lower (Q1) and upper (Q3) quartiles, the median and the mean (x) for each genotype. Bars with common letters do not show significant statistical differences as determined by one-way ANOVA with post-hoc Tukey's HSD test ($P<0.05$).

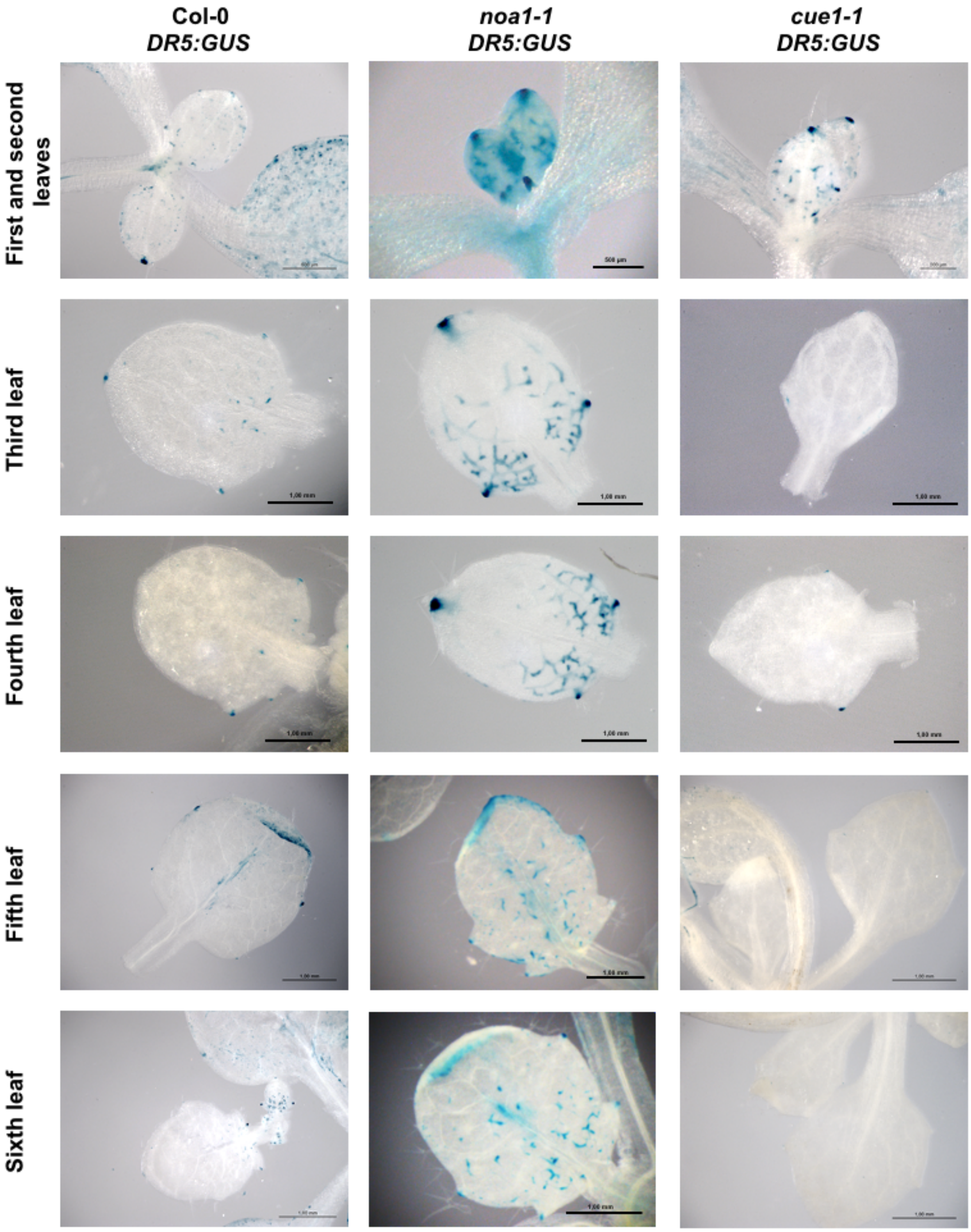

Supplementary Figure 3. Pattern of *DR5:GUS* expression in Col-0, *cue1/nox1* and *noa1-1*backgrounds, showing histochemical localization of ß-GUS activity during leaf morphogenesis in first and second leaves, third, fourth, fifth and sixth leaf of seedlings between 7- and 15-day-old. Scale bars, between 200 and 1000µm. At least 10 seedlings per genotype were analyzed all showing a similar expression pattern and a representative image was selected.

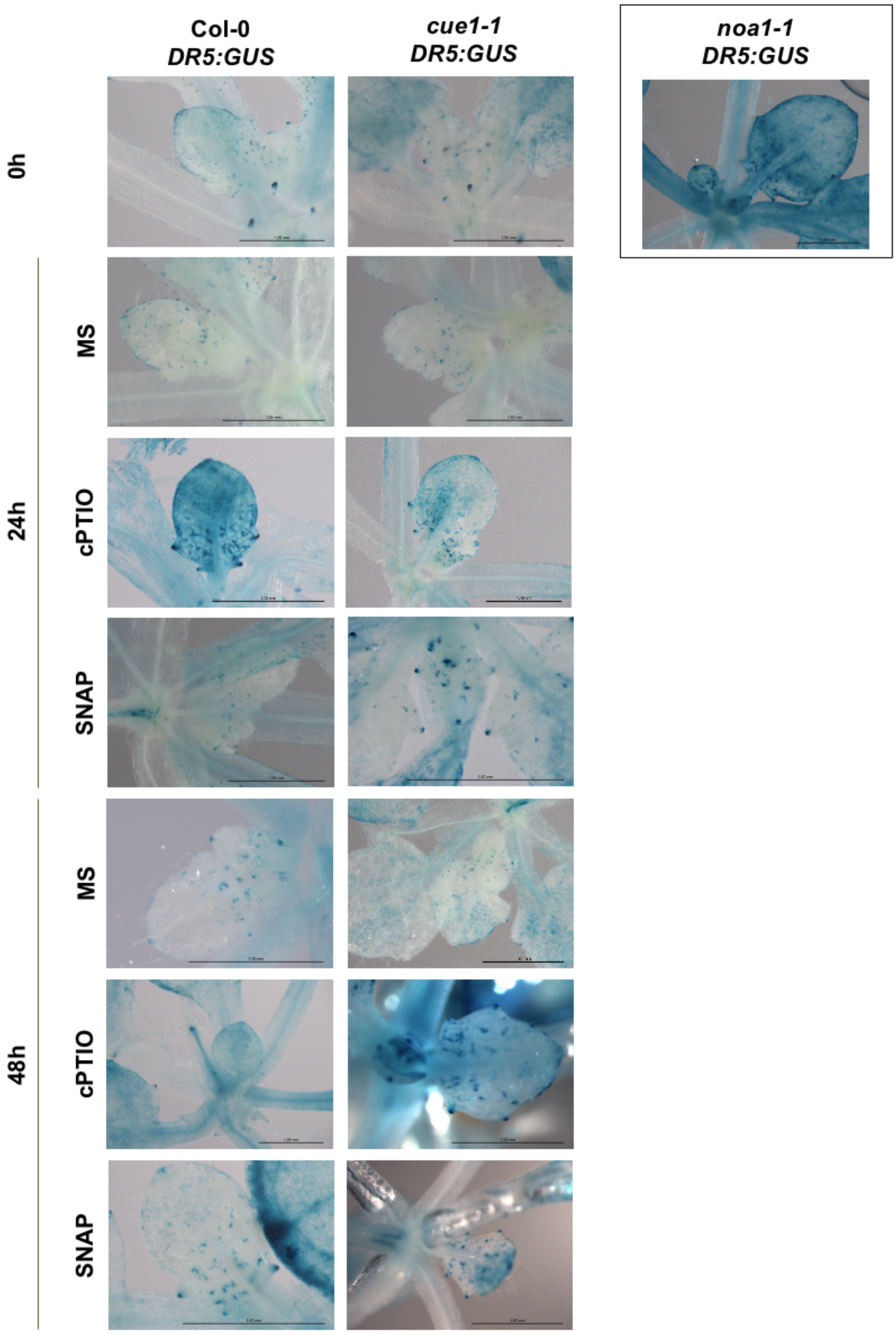

Supplementary Figure 4. Pattern of *DR5:GUS* expression in Col-0, *cue1/nox1* and *noa1-1*backgrounds, showing histochemical localization of ß-GUS activity during leaf morphogenesis of 15-day-old plants after treatments with the NO scavenger cPTIO (1mM) and the NO donor SNAP (300µM) during 24 and 48 hours. Scale bars, 1mm. At least 10 plants per genotype were analyzed all showing a similar expression pattern and a representative image was selected.

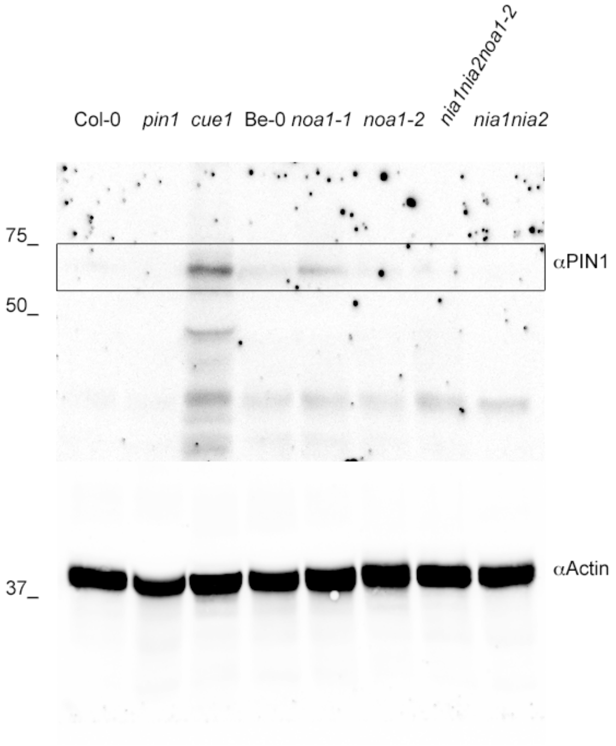
Col-0
pin1
cue1
Be-0
noa1-1
noa1-2
nia1nia2noa1-2
nia1nia2
75_
50_
37_
αPIN1
αActin

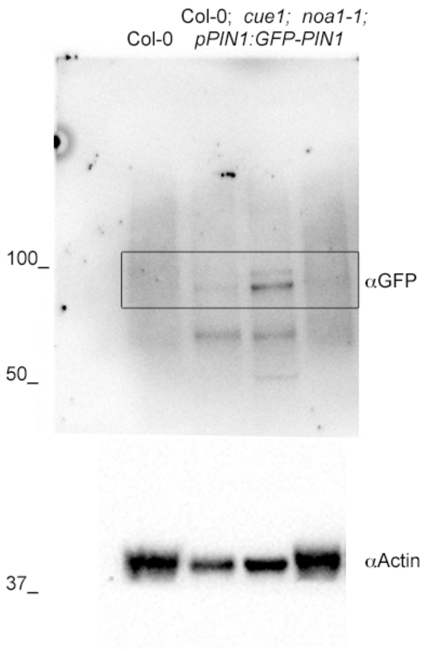
Col-0
Col-0; cue1; noa1-1;
pPIN1:GFP-PIN1
100_
50_
37_
αGFP
αActin

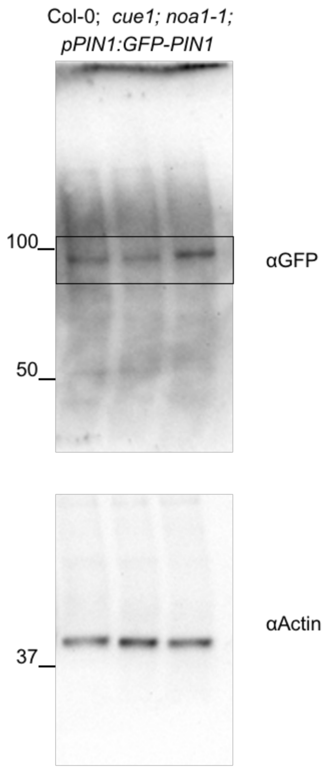
Col-0; cue1; noa1-1;
pPIN1:GFP-PIN1
100_
50_
37_
αGFP
αActin

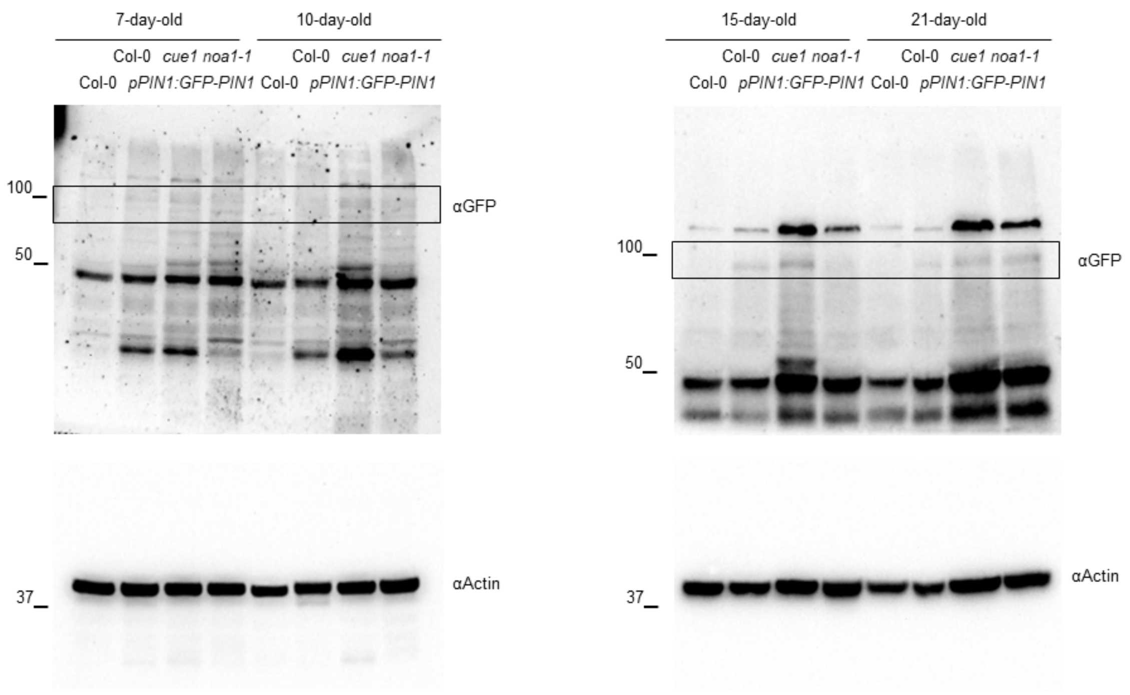

Supplementary Figure 5. Uncropped scans of the immunoblot results in Figure 6. Black boxes highlight lanes used.
